# Low-Carb and Ketogenic Diets in Type 1 Diabetes: Efficacy and Safety Concerns

**DOI:** 10.3390/nu17122001

**Published:** 2025-06-14

**Authors:** Emmanouil Korakas, Aikaterini Kountouri, Goran Petrovski, Vaia Lambadiari

**Affiliations:** 1 Department of Diabetes and Endocrinology, University College London Hospital, London NW1 2BU, UK; mankor-th@hotmail.com; 2 2nd Department of Internal Medicine, Research Institute and Diabetes Center, National and Kapodistrian University of Athens Medical School, 12462 Athens, Greece; katerinak90@hotmail.com; 3 Division of Endocrinology and Diabetes, Sidra Medicine, Doha P.O. Box 26999, Qatar; goran.endo@gmail.com

**Keywords:** type 1 diabetes mellitus, low-carbohydrate diet, ketogenic diet, glycemic control, insulin, ketosis, adolescents, pediatric

## Abstract

Despite advances in technology, the overall management of type 1 diabetes mellitus (T1DM) remains suboptimal. The idea of restricting carbohydrate intake to decrease glycemic spikes and insulin requirements has been revisited in recent years. After impressive results in the fields of type 2 diabetes (T2DM) and epilepsy, low-carbohydrate (LCD) and ketogenic (KD) diets have gained renewed interest as a possible treatment option for T1DM. In this narrative review, we discuss the available data regarding LCDs and KDs in both the adult and pediatric populations. Research data is still scarce, as most studies are short-term and show considerable heterogeneity in dietary composition and patient outcomes. In general, carbohydrate restriction enhances glycemic control by reducing postprandial glucose excursions, improving time-in-range, and lowering HbA1c, with conflicting effects on other parameters such as lipid profile and body weight. Adverse effects such as hypoglycemia and diabetic ketoacidosis are rarely reported, although some concerns have been raised regarding growth in children. The correct implementation of these diets requires a multidisciplinary approach by highly specialized healthcare professionals, who will address the medical, social, and psychological concerns that a restrictive diet entails. Large-scale and long-term studies are needed to provide more robust data before carbohydrate restriction can be widely applied to patients with T1DM.

## 1. Introduction

Type 1 diabetes (T1DM) is an autoimmune condition characterized by gradual pancreatic beta cell destruction, which eventually leads to insulin deficiency and hyperglycaemia [1]. Although ground-breaking technological advances over the last few years have increased the capabilities for continuous glucose monitoring and insulin administration, the overall management of T1DM remains suboptimal. In fact, only a modest 20% of children and 30% of adults achieve the guideline-suggested glycated hemoglobin (HbA1c) targets [2]. As newer basal insulins offer a generally steady absorption profile in basal–bolus regimes and, even more importantly, as more and more patients are using insulin pumps, it seems that the greatest challenge regarding euglycemia is the postprandial glycaemic spikes [3]. The introduction of the glycaemic index and the glycaemic load factors in determining the carbohydrate content of the meals has definitely facilitated insulin-dosing strategies, together with a better understanding of the role of dietary fat, protein, and fiber in carbohydrate absorption [4]. In this regard, programs such as the Dose-Adjustment for Normal Eating (DAFNE) program offer structured education regarding eating patterns, while allowing the patients to maintain a higher degree of flexibility with their diet [5]. However, a mismatch between carbohydrate absorption and insulin action continues to exist in the majority of cases.

As blood glucose excursions derive mainly from the input of carbohydrates from food, mainly in the form of sugars and starch, it would be rational to assume that reducing dietary carbohydrate could mitigate the miscalculation rate in pre-prandial insulin dosing and, therefore, reduce glycaemic variability and achieve more time in euglycemia. Indeed, the idea of low-carbohydrate diets as a treatment for T1DM is older than a century, and starvation diets were the mainstay of diabetes treatment in the pre-insulin era [6]. After the discovery of insulin, high-carbohydrate diets have been adopted by international guidelines as the suggested dietary regimen, mainly because insulin, with relatively simple adjustments, can ameliorate glycaemic excursion regardless of the amount of carbohydrate ingestion, at least in principle [7,8,9]. The indiscriminate consideration of dietary fat, regardless of its subtypes, as a risk factor for cardiovascular disease (CVD), along with the concerns for diabetic ketoacidosis (DKA) due to the poor understanding of the notion of nutritional ketosis, rendered high-carb diets as the leading dietary pattern in this patient population. However, in recent years, it seems that the older notions have come full circle, with low-carb or very-low-carb/ketogenic diets being considered a possible treatment choice for a number of autoinflammatory diseases. Currently, the main body of evidence, especially regarding ketogenic diets, comes from the field of epilepsy, where significant benefits have consistently been shown, even in drug-resistant forms [10,11,12]. In the metabolic domain, multiple randomized controlled trials (RCTs) have demonstrated that low-carb diets (LCD), meaning a total carbohydrate intake of <130 g/day or <26% of total energy intake (TEI), achieve substantial decreases in HbA1c along with improvements in lipid profile in patients with type 2 diabetes mellitus (T2DM) [13,14,15]. However, there is a serious paucity of data regarding T1DM, with few RCTs and evidence being mainly derived from small case series or observational studies. A systematic review from 2018 showed promising, but inconclusive results, mainly due to the high heterogeneity of the studies included [16]. In this narrative review, we aim to provide an overview of the literature data regarding low-carbohydrate and ketogenic diets in the management of T1DM and discuss their possible efficacy as a treatment choice, as well as safety and practical concerns.

## 2. Methodology for Literature Search

The studies mentioned in this review were retrieved by a computer search program using the PubMed, Scopus, and Web of Science electronic databases. The authors searched for scientific literature published in English up to February 2025. The search terms included combinations of keywords such as “type 1 diabetes mellitus”, “ketogenic diet”, “low-carbohydrate diet”, “glycemic control”, and “nutritional ketosis.” Studies were included if they involved the following: (1) involved human subjects with a confirmed diagnosis of type 1 diabetes mellitus (T1DM), (2) evaluated the effects of low-carbohydrate (<130 g/day) or ketogenic diets (<50 g/day), and (3) reported clinical outcomes such as HbA1c, insulin dose, glycemic variability, body weight, or safety events. We included RCTs, cohort studies, case series, and relevant case reports, given the scarcity of high-quality interventional trials in this field. Exclusion criteria were as follows: (1) studies involving only animal models, (2) studies with mixed populations (e.g., T1DM and T2DM) that did not report T1DM data separately, and (3) studies primarily focusing on other dietary interventions (e.g., Mediterranean, DASH) without a carbohydrate-restriction focus. The final reference list was generated based on relevance to the topic under discussion, with the aim of covering the available research data regarding the effects of carbohydrate restriction on T1DM outcomes in adults and children. We applied a narrative synthesis framework to group findings across several key sections, which included glycemic outcomes, adverse effects and safety profile, patient adherence and psychosocial aspects, special concerns for the pediatric population, and real-world feasibility and healthcare system integration. For the purpose of this review, LCD refers to <130 g/day and KD to <50 g/day carbohydrate intake. However, terminology across studies is inconsistent, limiting direct comparisons. Where necessary, individual definitions used in each study have been explicitly noted.

## 3. Carbohydrate Restriction in the Pathogenesis of T1DM: Pathophysiology Insights

Despite various and sometimes arbitrary definitions, it is now widely accepted that a low-carb diet is defined as a daily carbohydrate intake of <130 g, or 10–25% of TEI [6]. However, as the rationale behind limited carbohydrate intake in a state of insulin deficiency is rather unambiguous, the focus of interest has been shifted mainly towards ketogenic diets. By definition, the overarching target of the ketogenic diet is the increased formation of ketone bodies, namely β-hydroxybutyrate (BOHB), acetoacetate, and acetone. After a few days of implementation, such a diet results in a change in the body’s primary source of energy from glucose to ketone bodies. For such an effect to take place, daily carbohydrate intake needs to be reduced to <50 g, or <10% of TEI, with a concomitant increase in dietary fat consumption (70–80% of TEI) [17]. This leads to the so-called “nutritional ketosis”, namely, a concentration of β-hydroxybutyrate (BOHB) typically between 0.5 and 5.0 mmol/L [18]. A common misconception is that KD entails an unlimited protein intake; however, this can counteract the beneficial nutritional ketosis, as an abundant flux of amino acids can lead to increased glucose production through hepatic gluconeogenesis [19]. Therefore, total protein should not exceed 20% of the energy share.

The first step through which KD can possibly interfere in the pathogenesis of T1DM is the alteration of gut microbiota. In the study by Goffau et al. [20], the autoimmunization process in T1DM was associated with an increase in the population of Bacteroidetes and a simultaneous decrease in the abundance of lactate and butyrate-producing bacteria. The latter plays an important role in the protection of beta cells and, therefore, an increase in circulating butyrate levels can prevent autoimmunization [21]. However, apart from changes in the composition of gut flora, ketogenesis also exerts potent anti-inflammatory effects. Beta-hydroxybutyrate prevents the formation of several pro-inflammatory immune cells, such as the Th17 cells [22], and enhances the expansion of regulatory T cells (Tregs) [23], which play a crucial role in maintaining immune tolerance and suppressing excessive inflammation. T1DM is also characterized by increased oxidative stress, driven by the excessive production of reactive oxygen species (ROS) [24]. Both LCD and KD diets reduce AGE formation and have shown improvements in antioxidant defense mechanisms via Nrf2 activation in rats with diet-induced diabetes [25]. Other mechanisms also exist, such as suppression of the NLRP3 inflammasome through the suppressed activation of the NF-kB pathway and the reduction in ROS, though most research data derive from neuroinflammatory and neurodegenerative disorders [23,26].

Apart from functional alterations, restricted carbohydrate intake can also lead to quantitative modifications. In a study utilizing a streptozotocin-induced diabetic mouse model, a fasting-mimicking diet (FMD)—which promotes ketogenesis through nutrient deprivation—was shown to stimulate β-cell regeneration and to reverse hyperglycemia [27]. While these effects may be partially attributable to elevated ketone levels, given the overlapping metabolic features of fasting and ketogenic diets, it is important to note that FMD induces ketosis through a starvation-like state and should not be considered equivalent to a ketogenic diet. Moreover, definitive conclusions regarding the quantitative effects of ketogenic diets in the context of autoimmune T1DM cannot be drawn. This would require the use of the non-obese diabetic (NOD) mouse model, in which insulitis is the primary pathogenic mechanism leading to diabetes [28]. However, to date, no studies have investigated the impact of carbohydrate restriction in this specific animal model. In humans, notable findings were derived from a case report of a 19-year-old man with T1DM, on whom a paleolithic version of the ketogenic diet was applied [29]. Apart from improved glycaemic control within the first days, the patient demonstrated increased c-peptide concentrations after 10 weeks (2.2 ng/mL vs. 0.6 ng/mL), suggesting a restoration of his insulin secretory capacity, although the possible effect of the honeymoon period could not be excluded. However, inadequate insulin secretion is not the sole pathophysiological defect in T1DM. As many studies have shown in recent years, T1DM is also accompanied by a serious level of insulin resistance in all target tissues, namely the liver, the skeletal muscle, and the adipose tissue [30]. Especially at the level of adipose tissue, study data indicate impaired insulin-mediated suppression of plasma glycerol and non-esterified fatty acid (NEFA) levels during euglycaemic-hyperinsulinemic clamps, indicating a lower rate of insulin-mediated lipolysis suppression regardless of glycaemic control [30,31,32]. Potentially favorable hormonal actions of carbohydrate restriction have been studied in patients with obesity or epilepsy (but not with diabetes) and include an increase in anorexigenic hormones such as glucagon-like peptide 1 (GLP-1) and leptin, and suppression of the appetite hormone, ghrelin [33,34]. These actions, along with the decreased insulin requirements, can subsequently lead to weight loss and further enhancement in peripheral insulin sensitivity, an effect which is becoming increasingly important in light of the ever-growing prevalence of obesity among patients with T1DM [35]. In summary, while several mechanisms have been proposed to support a potential role for carbohydrate restriction in modulating immune and inflammatory responses relevant to T1DM, the evidence remains largely preclinical. Most of the data stem from animal models that do not fully recapitulate the autoimmune nature of human T1DM, or from isolated case reports. Therefore, no robust conclusions can currently be drawn regarding the efficacy of carbohydrate restriction to restore or preserve beta cell function in T1DM patients.

## 4. Carbohydrate Restriction in Adult Patients with T1DM

Research exploring LCD and KD in adult T1DM populations has shown some promising trends, particularly in terms of improved glycemic control, reduced insulin requirements, and decreased glycemic variability. However, the body of evidence is limited by substantial heterogeneity across studies, small sample sizes, and short durations of follow-up. In addition, some findings should be interpreted cautiously, as in some studies the data were self-reported and prone to recall or reporting bias, particularly regarding HbA1c and lipid parameters. In several observational studies and small trials, modest improvements in HbA1c, time-in-range (TIR), and hypoglycemia frequency have been reported. In the study by Nielsen et al. [36], 22 patients with T1DM (mean age 51 years) and high glycaemic variability adopted an LCD (70–90 g of carbohydrates daily) for a period of 12 months. At the end of the study period, the rate of hypoglycaemia was significantly lowered from 2.9 ± 2.0 to 0.5 ± 0.5 episodes per week, without an increase in blood glucose. HbA1c was significantly lowered from 7.5 ± 0.9% to 6.4 ± 0.8%, with a concomitant decrease in mean insulin requirements (21.1 vs. 12.4 units/day). A moderate but still significant reduction was also noted for triglyceride levels (TGs). In another study by the same group [37], where a LCD (<75 g of carbohydrates) was followed by 48 patients (mean age 52 years), mean HbA1c was reduced by 0.7% after 4 years of follow-up; when only the adherent patients were included in the analysis (48%). This decrease was as high as 1.3%, and it was accompanied by a significant increase in HDL levels, although TGs were not affected this time. Lipid effects have been rather neutral in other studies with LCD; in the study by Krebs et al. (mean age 44.6 years) [38], despite remarkable effects on HbA1c (8.2% vs. 8.9%) and daily insulin requirements (44.2 vs. 64.4 units/day) after 12 weeks, the low-carb diet (<75 g of carbohydrates) did not induce any important improvements in lipid parameters or body weight. The same neutral lipidemic effect was noted in the study by Turton et al. [39], where 16 patients (mean age 42.8 years) with poorly controlled T1DM were enrolled to participate in a 4-week control period following their usual diets and a 12-week intervention period following a LC diet (25–75 g/day of carbohydrates). Except for lipids, though, multiple aspects of glycaemic control were improved: mean HbA1c was reduced from 7.7% to 7.1%, mean insulin units were decreased from 65 to 49 units per day and, even more importantly, glycaemic fluctuations were mitigated, as expressed by an increased time in range (TIR, 74% vs. 59%) and decreased mean amplitude of glycaemic excursions (MAGE, 5.3 vs. 8.1 mmol/L). Similar results were shown in another small-scale study by Schmidt et al. [40], where 14 participants using sensor-augmented insulin pumps were allocated to either a low-carbohydrate diet (LCD < 100 g carbohydrate/d) or a high-carbohydrate diet (HCD > 250 g carbohydrate/d) for 12 weeks. Again, lipids were not affected; however, time spent < 3.9 mmol/L was less (1.9 vs. 3.6%), and glycaemic variability (assessed by coefficient of variation) was lower (32.7 vs. 37.5%) during LCD.

Evidence regarding stricter carbohydrate restriction on T1DM, namely ketogenic diets, points towards the same direction. In a retrospective study by Kleiner et al. [41], where 33 patients with T1DM voluntarily switched to a VLCD (<50 g of carbohydrates, 70% fats, 25% proteins and 5% carbohydrates) for 12 months, mean HbA1c decreased from 8.3% to 6.8% and there was a statistically significant decrease in the units of daily insulin (from 36.7 IU to 28.9 IU), with a concurrent reduction in from 54% to 24% in clinical level 2 hypoglycemia episodes. However, the small number of patients and the retrospective nature of the study are severe limitations that prevent causality associations. Leow et al. [42] recruited 11 patients with T1DM (mean age 36.1 years) who followed a ketogenic diet (<55 g of carbohydrates), and the duration of follow-up was 7 days. As expected, KD was associated with normal HbA1c levels and slight glycaemic fluctuations. Unfortunately, these benefits were accompanied by an unacceptably high median range of 0.9 daily episodes of hypoglycaemia. In fact, this might not have been a real adverse effect, but rather a physiological response to nutritional ketosis, as high ketone levels have a neuroprotective action and lower the hypoglycaemia threshold for neuroglycopenic symptoms and, therefore, hypoglycaemia awareness. LDL levels were also above the target range in 82% of the participants, an effect that was also noted in the questionnaire-based survey in 316 patients by Lennerz et al. (mean age 16 years, mean carbohydrate intake 36 ± 15 g) [43], where a ketogenic diet was also followed. Qualitative features of these lipid abnormalities, such as LDL particle size, were not addressed, which plays an important role in terms of cardiovascular risk, especially when taking into account the fact that increased saturated fat intake may increase small dense LDL particles, which are known to be more atherogenic. Glycaemic effect was nevertheless favorable, with the participant-reported reduction in HbA1c from pre- to post-KD being 1.45%. It needs to be taken into account; however, the results in this study were self-reported by the patients, with only a minority of them reporting lipid values or indexes of glycaemic variability. In the well-structured study by Ranjan et al. [44], which included 10 patients on an insulin pump (mean age 48 years), one week of KD resulted in a mean increase in TIR of 11%, a decrease in time spent in hypoglycaemia by 4.7%, and a concomitant decrease in glycaemic variability (mean SD 1.9 vs. 2.6 mmol/L). Such results were also shown in a retrospective case series of 26 adults with T1DM (mean age 35 years), which included both LCD and KD (mean carbohydrate intake of 63 g/day) [45]. Surprisingly enough, no KD or LCD interventions have mediated consistent and substantial decreases in the body mass index (BMI). One of the most consistent findings across studies of carbohydrate-restricted diets in T1DM is the reduction in total daily insulin requirements. This appears to result from multiple mechanisms. Primarily, lower dietary carbohydrate intake reduces postprandial glucose excursions, thereby decreasing the demand for bolus insulin. Furthermore, carbohydrate restriction may enhance insulin sensitivity, particularly through reductions in hepatic glucose output and improved suppression of lipolysis. Despite these reductions in insulin dose, though, meaningful changes in body weight were inconsistently observed. This suggests that the metabolic improvements conferred by LCD/KD may be independent of energy restriction and instead reflect altered substrate metabolism—favoring lipid oxidation over glycolysis—and modulation of appetite-regulating hormones. In patients with overweight or obesity, this shift may support moderate weight loss, but in lean individuals, unintentional caloric deficit may increase the risk of muscle catabolism or energy insufficiency if not properly addressed. Most of the data is summarized in the only available meta-analysis to date that examined the efficacy and safety of reduced carbohydrate diets in adolescents and adults with T1D [46]. This study included nine randomized controlled trials (RCTs) identifying four LCDs (<130 g/day), four moderate carbohydrate diets (MCD) (130–230 g/day or 26–45% TEI), and one study that used both an LCD and MCD. This meta-analysis found that the LCDs had no significant influence on HbA1c, but bolus insulin decreased (mean difference = −8.61 units/day) in participants with T1D who used a LCD. These findings could be attributed to the limited evidence searched, small sample sizes, and methodological differences in individual study designs. Even in the cases where results suggest an association between LCD and improved glycemic control, causality cannot be established due to the observational nature and small sample sizes of most studies.

Adherence and adverse effects are the two major concerns when implementing carbohydrate restriction, especially in the form of a ketogenic diet. Indeed, in the study by Krebs et al. [38], even after as soon as 12 weeks of a suggested daily carbohydrate intake of 50–75 g, the mean actual intake was 103 ± 22 g. In another cohort, after a mean follow-up of 4 years, only 48% of the patients remained adherent; statistically significant reductions in HbA1c; however, were achieved even by the partly adherent subgroup, with comparable improvements with the absolutely adherent group of participants [37]. Similarly, 9 of 20 people who volunteered for a KD intervention were excluded due to poor compliance with consumption of <55 g carbohydrate/day [42]. Adherence data are not reported in other studies, probably because of their short duration; for studies relying on self-reported data, HbA1c levels have been used as an index of adherence. The interpretation of adverse effects also requires caution. While some studies suggest reductions in hypoglycemia frequency, emerging data also point to unique risks. In one study, individuals on an LCD exhibited a blunted glycemic response to glucagon during insulin-induced hypoglycemia [47]. Fasting glucagon levels were higher after a week of LCD compared to a normal diet. This might lead to downregulation of glucagon receptors and, consequently, provide an explanation for this attenuated glycaemic response. Though only speculation, it seems that patients on LCD need higher doses of glucagon or alternative hypoglycemia rescue strategies to restore euglycemia. Although no significant increase in diabetic ketoacidosis (DKA) has been reported in adherent individuals under close supervision, this observation may not generalize to broader, less-monitored populations. The safety of KD in real-world T1DM management remains uncertain, especially when consistent ketone monitoring and clinician oversight are lacking. Education programs like DAFNE (Dose Adjustment For Normal Eating) may complement carbohydrate-restricted diets by teaching flexible insulin dosing and carbohydrate estimation. Their structured approach can mitigate risks like hypoglycemia or DKA when patients transition to lower-carb patterns, particularly if insulin-to-carb ratios change dramatically. Cardiovascular implications also warrant scrutiny. Effects of LCD/KD on lipid profiles have been inconsistent across studies, and in some cases, elevations in LDL cholesterol have been noted. While replacing saturated fats with polyunsaturated or monounsaturated alternatives can mitigate this risk, such dietary adjustments are not uniformly practiced. Polyunsaturated fat from vegetable oils seems to confer the greatest benefits in terms of CVD risk amelioration [48]. While short-term glycemic improvements have been consistently reported, the long-term cardiovascular and microvascular implications of sustained carbohydrate restriction in T1DM remain unclear. The current evidence lacks prospective trials assessing endpoints such as retinopathy, nephropathy, or cardiovascular events. Therefore, clinicians should weigh potential metabolic benefits against unverified long-term safety. The results of the main studies are summarized in Table 1.

## 5. Carbohydrate Restriction in Children with T1DM: Are There Any Special Concerns?

In pediatric populations, the evidence base for carbohydrate-restricted diets is extremely limited and primarily consists of case reports and small observational studies, often involving children with comorbid epilepsy. In c case report by McClean et al. [49], a 4-year-old boy with myoclonic-astatic epilepsy and T1DM followed a ketogenic diet for 6 years, achieving normal HbA1c values without DKA or hypoglycaemic episodes during follow-up. In other similar cases, the implementation of a KD for 15 months [50] and 10 months [51], respectively, led again to HbA1c values within the target range, without any serious adverse effects. Even in the absence of epilepsy, KD achieved substantial amelioration of glycaemic control, which even led to the utter discontinuation of insulin in a pre-pubertal child [52]. However, such insights from case reports cannot be extrapolated. More structured studies raise significant concerns. For instance, in a small cohort reported by de Bock et al. [53], which included six children with T1DM who followed a ketogenic diet, although HbA1c improved, poor growth occurred in three of the six cases, which was combined with a high frequency of hypoglycaemic episodes in two cases. Interpretation of growth data in pediatric cohorts must be cautious, as existing studies are observational, underpowered, and subject to confounding. Hence, definitive conclusions regarding causality between LCD/KD and growth outcomes cannot be drawn. In the online survey by Lennerz et al. [43], a modest though statistically significant decrease in height percentile from 0.41 at diagnosis to 0.2 at time of data collection was reported in 34 children, but whether this growth deceleration occurred during the diet or was already evident before was not possible to clarify. In addition, the magnitude of growth impairment resembled the typical trend observed in large T1DM registries, which is ascribed to poor glycaemic control and reduced circulating insulin levels [54,55,56]. It is well-known that insulin is involved in growth hormone response and has a favorable effect on IGF-1 release from the liver [53]. Therefore, it seems that relative insulin deficiency, as a result of carbohydrate restriction, is the main culprit mechanism for growth retardation, especially in the pediatric T1DM population where insulin levels are already low or undetectable [57]. It is vital that unintentional caloric restriction, which frequently takes place during carbohydrate restriction, is avoided in these patients; protein intake should be optimized, adjusted for age, pubertal stage, and activity level, while supplementation with micronutrients like vitamin D, calcium and magnesium could be offered as long as the strict dietary regime is instituted, to prevent bone health issues. Given these potential risks and the lack of high-quality, long-term pediatric data, the use of low-carbohydrate or ketogenic diets in children with T1DM should be regarded as experimental and approached with significant caution. Multidisciplinary supervision, rigorous nutritional planning, and close monitoring of growth, metabolic status, and psychosocial well-being are mandatory in any case where such diets are considered. Until more robust evidence becomes available, these approaches cannot be recommended outside of a research setting.

## 6. Beyond Metabolic Control: Challenges and Concerns

Low-carbohydrate and ketogenic diets limit the daily intake of foods such as wholegrains, starchy vegetables, legumes, fruits, and dairy products [58,59]. All these food categories provide invaluable micronutrients such as vitamins, electrolytes, and fiber, with well-established cardiovascular and metabolic benefits. Concerns about calcium levels have been raised in a pediatric population following carbohydrate restriction [50,56], and iron deficiency has been a common finding in low-carb, high-fat diets due to hepcidin-independent reduction in duodenal iron absorption [60]. In addition, carbohydrate restriction can frequently lead to inadvertent caloric restriction and, thus, a substantial energy deficit [38]. The subsequent weight loss might be an obvious benefit for patients with T1DM and obesity, but it could increase the risk of ketoacidosis in lean individuals or lead to growth deceleration in children. Caution is also necessary when increasing the fat content of the diet to replace the carbohydrate deficit, as many patients eventually increase saturated fat intake even as high as 11% of their daily fat intake, with detrimental implications for their cardiovascular health [42,61]. In general, close monitoring and strict dietitian review are necessary after the institution of a low-carbohydrate diet to evaluate any possible nutritional deficiencies and advise on foods and products for substitution or supplementation.

Apart from primarily medical concerns, social issues might also arise. Low-carbohydrate and ketogenic diets are restrictive diets, and the psychological implications might be serious in patients with T1DM who already have a higher frequency of eating disorders [62]. Diabulimia, namely the practice of skipping or reducing insulin doses to achieve weight loss, is common among people with T1DM and might be further exacerbated by restrictive dietary patterns [63]. In addition, as food is an integral part of many social and recreational activities, patients, especially children, might find an LCD to be socially isolating [64]. These concerns are not limited to children and adolescents; adult patients, as well as the parents of children with T1DM on carbohydrate restriction, also face challenges. Increased food preparation time, lack of available resources, and limited variety of choices are important considerations for many patients or carers and can seriously hamper compliance and adherence [64]. Religious and cultural beliefs may also hinder the institution of a “classic” ketogenic diet, requiring alternative sources of fat such as plant proteins [65]. Obviously, such factors undermine adherence, especially when patients do not receive adequate support from their treatment team. In the questionnaire-based survey mentioned before, participants reported high levels of overall satisfaction with their diabetes management team. However, a total of 27% had concealed the fact that they were following a KD because they were afraid of being judged or because they were not confident about the physicians’ interest or familiarity with ketogenic diets [41]. Of those who discussed their dietary choice with their treating physicians, more than half of them (51%) complained that they did not feel supported in following carbohydrate restriction. The reason for this phenomenon, which is widely reported in clinical practice, is the healthcare providers’ concerns regarding adverse effects. In addition, the lack of healthcare resources often leads to dietitian-only appointments, with limited support from a diabetes specialist who will provide a detailed plan on insulin adjustments and sick day rules.

Several practical considerations arise regarding the impact of carbohydrate restriction on hybrid-close loop systems. HCL algorithms are typically optimized for standard carbohydrate-based meals and may not automatically adjust to the reduced postprandial glucose excursions seen with low-carb or ketogenic diets. Manual adjustment of insulin-to-carbohydrate ratios, correction factors, and basal rates may be required. Furthermore, lower overall insulin requirements associated with carbohydrate restriction can alter system responsiveness, potentially affecting algorithm performance. These limitations highlight the importance of an individualized approach and comprehensive patient education when implementing such dietary regimes in patients on insulin pumps or HCL systems.

In general, dietary patterns have been mainly studied in patients with T2DM, where not only carbohydrate restriction, but also other approaches such as Mediterranean Diet (MD) and Dietary Approaches to Stop Hypertension (DASH) diet have well-established benefits both in terms of glycemic control and in the prevention of micro- and macrovascular complications [66,67,68]. On the other hand, nutritional management in T1DM remains challenging. In a cross-sectional study with 97 patients, the odds of having HbA1c < 7.5% increased in children with high adherence to MD (R: −0.245), while there was a positive correlation between TIR and adherence score (R: 0.200) [69]. TIR was also improved in another cohort of 20 adolescents following MD, but no similar results were demonstrated for HbA1c, and the slight decrease in total daily insulin dose was marginally significant [70]. Similarly, the DASH diet or its specifically modified for diabetes (DASH-D) version has shown some favorable results in glycemic variability, but data are generally scarce [71]. The American Diabetes Association (ADA) guidelines do not dictate any specific pattern as a «gold standard» for T1DM, as existing data do not support a specific macronutrient pattern. Instead, they emphasize the increased consumption of high-fiber sources of carbohydrate and the avoidance of foods and beverages rich in sugar or artificial sweeteners, along with the substitution of saturated fats with mono- or polyunsaturated ones. Ketogenic diets are discouraged only in patients on SGLT-2 inhibitors, which are used not only in T2DM but sometimes also in T1DM off-label to improve glycemic variability. Genetic and metabolomic variants, or the gut microbiota, can significantly influence a patient’s response to different diets, which is the reason why an individualized approach is necessary, along with education and behavioral therapy.

Taking these concerns into account, it becomes evident that the application of carbohydrate restriction in T1DM requires a multidisciplinary approach. Healthcare providers need to be up-to-date and upskilled, so that they feel confident with dietary management and are able to provide alternatives according to the patients’ targets and wishes. A thorough discussion regarding carbohydrate quality, alternative sources of fat and protein, meal structure, and insulin boluses needs to be conducted [72,73]. The use of continuous glucose monitoring (CGM) systems and ketone meters is necessary to prevent hypoglycaemia and diabetic ketoacidosis and will guarantee better physician support and an increased sense of safety for both them and their patients. A high frequency of follow-up, with intervals as narrow as 2 weeks, is of paramount importance, especially in the case of ketogenic diets, which are generally considered short-term interventions, as studies in patients with T2DM have shown [74,75]. In the pediatric population, the standard T1DM follow-up needs to be stricter and more targeted with items specific to the implications of carbohydrate restriction, such as anthropometric parameters, micronutrient deficiencies, and lipid profile in shorter intervals [76,77]. It is clear that the “one-size-fits-all” approach is obsolete; as it is the case with any dietary intervention, carbohydrate restriction needs to be individualized to tailor the specific needs of the patient and provide them with a plan that will feasible and relatively simple to follow, as adherence is the most crucial factor to witness any tangible benefits.

## 7. Conclusions

This review presents the synthesis of available evidence, which, while suggestive of clinical benefit from LCD/KD in certain adult T1DM populations, remains limited by the small sample sizes, short durations, and lack of uniform dietary definitions across studies. The overall strength of evidence is moderate to low, particularly regarding long-term efficacy and safety. A cautious interpretation is warranted, especially in light of the observational design of most studies and self-reported outcomes. In conclusion, although reductions in hypoglycemia and diabetic ketoacidosis have been reported in selected settings, these outcomes should be interpreted with caution given the limited generalizability and the need for intensive monitoring. Moreover, concerns around lipid alterations, growth impairment in children, nutritional adequacy, and psychosocial well-being remain unresolved and warrant further investigation.

Given these limitations, LCD/KD should not be recommended as a routine dietary approach for T1DM. Their use may be cautiously considered in highly motivated, well-supported adult patients with structured education and access to continuous glucose and ketone monitoring. Even in such cases, interventions should be time-limited and implemented under the close surveillance of a multidisciplinary team including medical, dietary, and mental health professionals. Large-scale, longer-term randomized controlled trials need to be conducted to confirm these findings. These would include longitudinal studies specifically designed for pediatric and adolescent populations, the effects on mental health, eating behaviors, and social participation. In adults, increased meal planning demands and household dynamics (e.g., cooking for multiple dietary needs) also need to be addressed in real-world settings, as such factors can hamper the adherence of both them and their children. The ideal range of ketosis necessary to achieve benefits in a safe manner needs to be meticulously investigated, and this is closely tied to the use of advanced diabetes technologies, including continuous glucose monitoring (CGM) and home ketone testing. These tools are not universally accessible, especially in healthcare systems with limited resources, and, thus, socioeconomic disparities may widen if such regimes are promoted without addressing affordability and availability. Finally, if longer-term interventions are to be considered, the evaluation of macro- and microvascular complications is of utmost importance, and would also entail advanced lipid testing (e.g., ApoB, LDL particle size). Until such data is available, the widespread application of these diets in the T1DM context cannot be endorsed. Individualized and evidence-based dietary strategies remain the cornerstone of diabetes nutrition therapy, with patient safety and long-term health outcomes as guiding principles.

## Figures and Tables

**Table 1:** Effects of low-carbohydrate and ketogenic diets in adults with type 1 diabetes mellitus. HbA1c, glycated hemoglobin; TIR, time in range; TBR, time below range; HDL, high-density lipoprotein cholesterol; CV, coefficient of variation; VLCD, very-low-carbohydrate diet; LCD, low-carbohydrate diet; KD, ketogenic diet; LDL, low-density lipoprotein cholesterol; TC, total cholesterol; TGs, triglycerides; GMI, glucose management indicator.

Sample Size/ Mean Follow-Up/ Diet [Reference]	Mean Age (y)	BMI (kg/m^2^)	HbA1c (%)	TIR (%)	Insulin (U/d)	Hypoglycemia	Body Weight (kg)	GV	Lipids
16 patients/ 16 weeks/ LCD/KD [39]	42.8	31.8	−0.6%	16%	−16	≠	−2.4	−0.8 SD	≠
48 patients/ 4 years/ LCD [37]	52	25.9	−1.8% (adherent)	n/a	≠	n/a	n/a	n/a	+12% HDL ↑ TC/HDL
10 patients/ 12 weeks/ LCD [38]	44.6	27.5	−0.7%	n/a	−22.2	n/a	≠	≠	≠
22 patients/ 12 months/ LCD [36]	51	−	−1.1%	n/a	−8.7	−2.4 episodes/week	↓	n/a	−25% TGs
14 patients/ 12 weeks/ LCD [40]	44	25	≠	n/a	≠	−1.7% TBR	−2.0	CV −4.8%	≠
33 patients/ 12 months/ VLCD [41]	41.6	23.9	−1.5%	≠	−7.8	−30% Level 2	−2.9	CV −10%	−14.1% LDL
11 patients/ 7 days/ KD [42]	36.1	23.4	Normal levels	74 ± 20%	n/a	0.9 episodes/day	n/a	CV 26 ± 8%	LDL: 5.5 mmol/L TC: 7.9 mmol/L
10 patients/ 2 weeks/ KD [44]	48	24.8	≠	+11%	−10.1	−4.7%	≠	−7.7% CV	≠
26 patients/ 55 weeks/ LCD/KD [45]	35	27	−2% −0.8% GMI	+18%	−13	−26 min over 2 weeks	n/a	n/a	n/a
